# Editorial: Organic waste and by-products: derived compounds as functional agents from fermentation processes

**DOI:** 10.3389/fnut.2024.1453879

**Published:** 2024-09-02

**Authors:** Gheorghe Adrian Martău, José Pablo López-Gómez, Lavinia-Florina Călinoiu, Teodora Emilia Coldea, Elena Mudura, Dan Cristian Vodnar

**Affiliations:** ^1^Institute of Life Sciences, University of Agricultural Sciences and Veterinary Medicine, Cluj-Napoca, Romania; ^2^Faculty of Food Science and Technology, University of Agricultural Sciences and Veterinary Medicine, Cluj-Napoca, Romania; ^3^Leibniz Institute for Agricultural Engineering and Bioeconomy, Potsdam, Germany; ^4^AINIA, Paterna, Valencia, Spain

**Keywords:** organic waste valorization, by-product management, solid-state fermentation, submerge fermentation, circular economy, bioprocess engineering

Societal attitudes toward waste have shifted in recent years, emphasizing waste and by-product valorization. The surge in organic materials production from various vegetable sources has prompted international organizations to advocate for reimagining waste as valuable secondary resources rather than pollutants. This evolving framework offers opportunities for converting large volumes of waste and by-products into energy or valuable compounds *via* fermentation processes (i.e., aroma compounds: vanillin; vitamins: B12; mycoproteins: essential amino acids; organic acids: lactic acid, etc.) (1–4).

**Figure 1:**
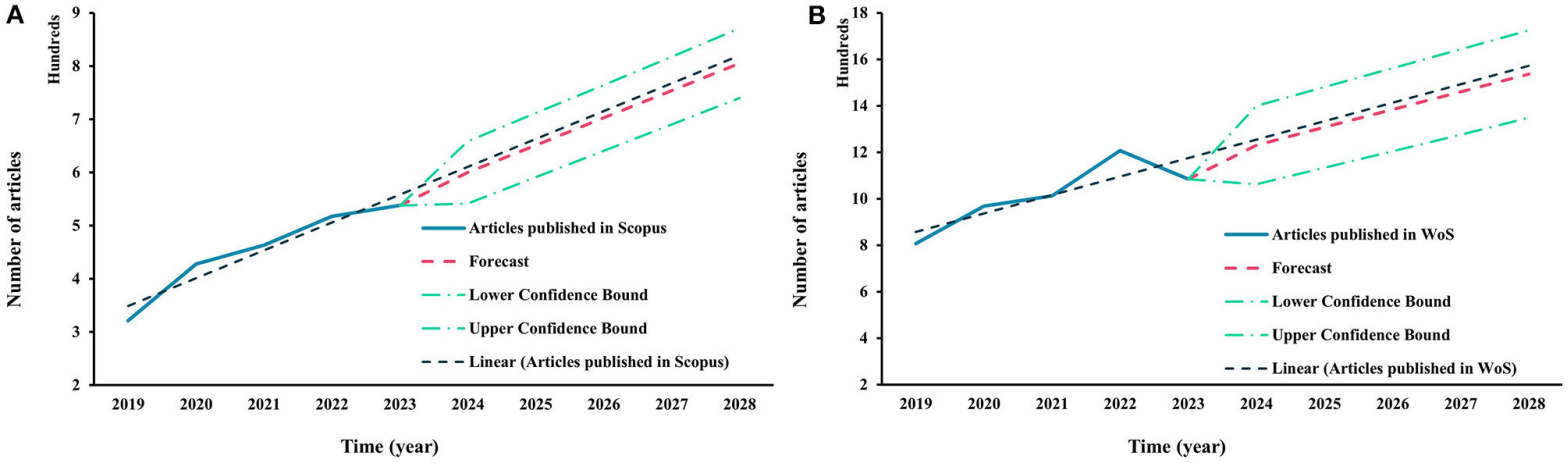
Research articles published in the last 5 years on Scopus **(A)** and the Web of Science™ **(B)** database, focused on “*fermentation*” and “*by-product*,” or “*fermentation*” and “*organic waste*,” and trend until 2028.

In the past 5 years, over 2260 research articles were published on Scopus (SP) and 5080 on the Web of Science™ (WOS) database, that have focused on “*fermentation*” and “*by-product*,” or “*fermentation*” and “*organic waste*,” highlighting an increasing trend in utilizing these by-products or organic waste substrates. In addition, considering the last 5 years, the number of published articles, and the Forecast function, it was possible to predict the number of articles published until 2028 based on existing values. The trendline was calculated considering articles published in the last 5 years. The Trend function returns values along with a linear trend. A confidence interval was estimated using interval values from 2019–2023. Its lower and upper bounds defined the interval from 2023 until 2028. The confidence interval was expressed as a percentage (95%). According to data analysis from the SP and WOS databases, the number of articles forecast may reach 805 and 1536 by 2028. Figure 1 shows a constant forecast and trendline that will increase in the following years. Considering these factors, the results highlight the research interest in waste and by-product management and the potential number of articles that could be generated in this research area.

This Research Topic presents current knowledge and research trends in food waste and by-products, highlighting management strategies at all food production levels and integrating fermentation technologies. Reintegrating by-products or organic waste into productive systems promotes sustainability and innovation in biotechnology, aiding in sustainable development goals and addressing resource and waste management challenges. The organic waste fraction includes materials from households, restaurants, various businesses, and yard waste (5). Numerous fermentation processes on these organic wastes have been extensively studied in recent years, including solid-state fermentation, aerobic and anaerobic liquid fermentation, and photo-fermentation (6, 7). These processes often involve treatment methods, such as enzymatic hydrolysis, chemical hydrolysis, anaerobic digestion, and thermal treatments, to enhance the compound bioavailability present (8, 9). Agro-industrial residues like fruit pomace, wheat bran, rice bran, hull-less pumpkin oil cake, hemp, and flax oil cake are extensively utilized in leveraging by-products as functional agents or substrates for fermentation processes. These materials, generated in significant quantities by the food industry, can be repurposed as substrates for energy production or for synthesizing valuable compounds [(10), Precup et al.]. In addition, the valorization of waste and by-products is of interest regarding the content of bioactive compounds present in these organic fractions. Faiza et al. published an article that aimed to extract and characterize polyphenols from corn by-products by conventional and green extraction techniques, obtaining promising results. Also, global warming directly impacts plant quality and food digestibility. To address this, alternative technologies that integrate the production of crops adapted to the changing climate are recommended. This is crucial to safeguarding food security and human nutritional health (Huang et al., Liu et al.).

Agro-industrial food residues are nutrient-rich solid substrates that facilitate nutrient absorption and support biomass growth in various fermentation systems, enhancing the efficiency and yield (11). This fermentation process exploits these substrates to cultivate multiple microorganisms, including fungi, bacteria, and yeast. For instance, *Aspergillus niger* and *Trichoderma reesei* are extensively utilized in solid-state fermentation for the production of vital industrial enzymes such as cellulase, lipase, amylase, and pectinase. Different cereal by-products produce glycosidic enzymes, while fruit pomace is employed for pectinolytic enzymes. These enzymes play crucial roles in numerous biotechnological applications, including biomass degradation, biofuel production, and food processing (12, 13). *Bacillus* spp. and *Yarrowia* spp. are prominent in submerged fermentation processes aimed at the biosynthesis of organic acids, like lactic, citric, and gluconic acid, which are valuable in the food, pharmaceutical, and chemical industries (14, 15).

In summary, given the importance of the challenges, we should anticipate a significant increase in research on solid and submerged fermentation that will integrate waste and by-products. Both research and policies will align with this goal.
